# Demographic and clinical characteristics of frequent users of emergency medical services in Türkiye

**DOI:** 10.55730/1300-0144.6343

**Published:** 2026-04-06

**Authors:** Burak BEKGÖZ, İshak ŞAN

**Affiliations:** Department of Emergency Medicine, University of Health Sciences, Ankara Bilkent City Hospital, Ankara, Turkiye

**Keywords:** Emergency medical services, frequent users, ambulances

## Abstract

**Background/aim:**

Overuse of emergency medical services (EMS), particularly unnecessary ambulance requests, places considerable strain on limited healthcare resources and can delay responses to urgent cases. The present study analyzes the demographic characteristics, diagnoses, urgency, and hospital transfer status of frequent users of EMS in Türkiye.

**Materials and methods:**

Included in the study were 41,113 patients who requested EMS four or more times within a year (defined as frequent users for the purpose of the study), excluding interhospital transfers. Data were accessed from the Emergency Medical Services Automation System (ASOS) database, which contains details of patient demographics, diagnoses, triage codes, reasons for calls, interventions, time spent at the scene, and hospital destinations.

**Results:**

During the study period, frequent users accounted for 282,143 of all ambulance assignments. The mean age was 59.0 ± 23.5 years; those aged ≥65 and above comprised the largest group (50.2%); 50.2% were female, and the mean number of EMS utilizations per patient was 13.4 ± 26.1. Nontraumatic medical reasons accounted for 92.4% of the total ambulance requests, with dyspnea, COVID-19, and nausea/vomiting being the most common in adults, and epilepsy, fever, and nausea/vomiting in pediatric patients. Ambulance teams spent an average of over 50 min per case, and most requests were made between 08:00 and 15:59.

**Conclusion:**

Requests for ambulances in non-emergency situations may contribute to delays in the care of other patients. Further research is needed to explore the underlying reasons for frequent EMS utilization and to develop targeted strategies, such as improved chronic disease management and enhanced access to outpatient and home care services, and to optimize EMS resource allocation and patient outcomes.

## 1. Introduction

Emergency medical services (EMS) were initially established to respond to emergency situations; however, they have recently been expanded to provide services to those with chronic diseases as well [1]. EMS usage rates are increasing steadily in line with the rising population. A study conducted in Australia found that ambulance callouts increased by 29.2% between 2008 and 2015 [2]. While population growth accounts for a considerable proportion of this increase, there are other factors that play a role, including demographic changes (e.g., the aging population), the increased prevalence of chronic diseases and comorbidities, changes in disease patterns (e.g., acute exacerbations of chronic diseases), and the number of patients exceeding the bed capacity of hospitals [3].

The overburdening of EMS is a global issue, although there is no clear consensus on what constitutes “frequent use”. Patients seek EMS when they cannot meet their own health or social care needs, and when they are unable to access appropriate services due to such reasons as living alone [4]. Repeated ambulance requests are one form of frequent EMS utilization. One previous study reported a repeated ambulance use rate of 16% [3], while a review study reported repeated ambulance use in the range of 0.2–23%, constituting 1.4–40% of all ambulance trips [5]. A review of the literature indicates that there is no universally accepted definition of frequent ambulance use, with broad variations across countries. Previous studies have not achieved standardization in this regard, with frequent ambulance defined as more than three calls per month in some countries, and more than 10 calls per month in others [6].

The literature review further revealed that individuals who make frequent requests for ambulance services often face vulnerabilities in terms of their health status, social conditions, limited access to alternative care pathways, and complex physical and psychological needs. Unless these issues are adequately addressed, patients will continue to be a burden on ambulance services [7]. EMS, like other healthcare branches, have limited resources. When an ambulance team is assigned to a nonurgent case, more urgent cases may be missed [3]. In countries with a high patient burden, such as Türkiye, repeated ambulance requests overburden the system, making it difficult to ensure the timely provision of services to patients with time-sensitive conditions. The present study investigates the demographic characteristics, diagnoses, urgency levels, and hospital transfer statuses of patients who make frequent use of EMS.

## 2. Materials and methods

As there is no established cutoff value or standardized definition for frequent use in the literature, we sought to develop a context-specific standard tailored to our country. In the EMS unit from which our data were obtained, ambulances were called out 563,011 times to a total of 169,248 patients annually, equating to a mean number of ambulance assignments per patient of 3.3. Based on this figure, patients of all ages who made use of EMS 4 or more times in a year were considered frequent users in the present study (not including between-hospital transfers). The patients’ diagnoses were categorized according to the International Classification of Diseases-10 (ICD-10).

Data for this retrospective study were obtained from the database of a provincial EMS unit that assigned ambulances to an average of 560,000 cases annually between 2018 and 2022. No appropriate sample size was calculated for the study, as the data of all 41,113 frequent users of EMS were included in the study. Ambulances were dispatched to these frequent users on 282,143 occasions, and the analyses were conducted based on the total number of ambulance dispatches. Recurrent calls were matched using the patients’ Republic of Türkiye identification numbers.

The study protocol was approved by the Ethics Committee of Bilkent City Hospital (No. TABED 1-24-365).

The study data were obtained from Emergency Medical Services Automation System (ASOS) database of the Turkish Ministry of Health. The ASOS database records information on age, sex, diagnosis and triage code, reason for the call, interventions by the ambulance team, and the total time spent at the scene and for transportation of the patient to hospital for each patient, as well as the team dispatch and arrival times, case location, and the medical data and demographic characteristics of the patient.

### 2.1. Limitations of data sources

Patient information is recorded in the ASOS database by the 112 Emergency Call Center, and appropriate teams are dispatched accordingly. Upon arrival at the scene, ambulance teams assess the patient and enter demographic data, vital signs, preliminary diagnoses, and the destination hospital into the ASOS system. After this data has been uploaded, it cannot be modified. The ASOS system servers are hosted by the Ministry of Health and cannot be accessed externally, making it a secure and well-protected system with a very low likelihood of data loss.

Patients with multiple calls and ambulance dispatches were matched using their Republic of Türkiye Identification Numbers to prevent any potential misclassification. Consequently, the likelihood of data loss or mismatch in this context is also very low.

### 2.2. Statistical analysis

All data were analyzed using IBM SPSS Statistics 27.0 for Windows (IBM Corp., Armonk, NY, USA). In addition to descriptive statistical methods (frequency, percentage, mean, standard deviation, median, min-max), the chi-square (χ^2^) test was used for the comparison of qualitative variables. The compliance of the data to normal distribution was evaluated with a Kolmogorov–Smirnov test, skewness and kurtosis values, and graphical methods (histogram, Q-Q plot, stem-and-leaf plot, boxplot). The independent samples t-test was used to evaluate quantitative data that complied with normal distribution. The statistical significance level was accepted as α = 0.05.

Multivariable logistic regression models were constructed to identify any variables independently associated with high call frequency, hospital transport decision, and prolonged engagement time. In all models, sex, age group, reason for call, and primary diagnosis categories were included simultaneously. Results were reported as adjusted odds ratios (ORs) with 95% confidence intervals (CIs) and p-values. The p-values presented in the regression tables were derived from the Wald test for the corresponding coefficients in the multivariable logistic regression.

## 3. Results

A total of 41,113 patients requested an ambulance 4 or more times during the study period, and ambulances were dispatched 282,143 times. Of the patients, 50.2% were female, the mean age was 59.0 ± 23.5 years. Those aged ≥65 years constituted the largest patient group (50.2%), while the pediatric age group had the lowest number of patients (5.5%). While most patients (98.5%) were citizens of the Republic of Türkiye (Table 1), there were statistically significantly more foreign-born patients in the pediatric age group than in the adult age group (5.7% vs. 1.3%, p < 0.05).

The mean number of EMS callouts among the frequent users was 13.4 ± 26.1. The number of EMS utilizations per patient in the pediatric age group was statistically significantly higher than that of the adult age group. The mean team intervention time was 911.1 ± 712.5 s per case, and the mean case occupation time was 3025.3 ± 1803.6 s. Ambulance teams traveled an average of 18.1 ± 9.7 km per case. While the intervention period was statistically significantly shorter in the pediatric age group compared to the adult age group (857.6 ± 654.2 s vs. 914.2 ± 796.3 s, p < 0.05), the occupation time (3476.1 ± 2285.3 s vs. 2998.9 ± 1767.9 s, p < 0.05) and the distance covered (25.1 ± 24.2 km vs 17.7 ± 19.3 km, p < 0.05) were statistically significantly longer (Table 2).

The sample most frequently used EMS ambulances for non-traumatic medical reasons (92.4%), with the predominant diagnoses being dyspnea (9.4%), COVID-19 (5.9%), and nausea and vomiting (5.0%). In the adult patient group, the most common diagnoses were dyspnea (9.7%), followed by COVID-19 (6.1%), and nausea and vomiting (5.0%). In patients <18 years, the most common diagnoses were epilepsy (12.7%), fever (6.1%), and nausea and vomiting (5.5%). In the multivariable model evaluating hospital transport decisions, dyspnea, nausea–vomiting, falls, COVID-19, hypertension, fever, and chest pain in particular were found to be independently associated with hospital transport. No statistically significant independent association was observed for the categories of pain and epilepsy (Table 3).

The patients were most frequently transported to hospital (76.0%), while only 1.1% were treated at the scene and 22.9% refused transportation to hospital. The patients were most frequently transported to a tertiary hospital (60.9%). Adult patients were most frequently transported to tertiary (61.5%) and secondary (24.3%) hospitals, while pediatric patients were most frequently transported to tertiary (51.4%) and university (31.0%) hospitals (Table 4).

Frequent EMS utilization was most common in 2019 (22.1%), and January and March (8.7%) were the months with the most frequent ambulance requests. Ambulances were requested most frequently between 08:00 and 15:59 (41.8%), accounting for 50.9% and 41.3% of the callouts for pediatric and adult patients, respectively. The pediatric age group used EMS most frequently in March (9.2%) and December (9.1%), while the adult age group used EMS most frequently in January (8.7%) and March (8.7%) (Table 5).

## 4. Discussion

A study of frequent EMS users in the United Kingdom reported that more than half of the patients were female and the mean age was 57.6 years [8]. Another study evaluating frequent EMS users reported most patients (42.9%) to be older adults [9]. In our study, similar to the literature, the mean age was 59 years, most of the patients (50.2%) were ≥65 years old, and more than half were female.

In the present study, the pediatric age group was smaller than the adult age group, although the number of ambulance requests per patient was statistically significantly higher for the pediatric patients. In contrast, a study conducted in the United States found that frequent users were least common within the pediatric age group (0–15 years) and that patients of all other age groups, primarily those aged 45–54 years, were significantly more likely to be frequent users [10]. In the present study, the ≥65 years age group contained the highest number of patients. There has been no study to date evaluating the mean number of EMS utilizations per patient categorized according to age group. The higher mean number of EMS utilizations per patient in the pediatric group in our study may be attributed to the differences in family structures between countries. We believe that a small, specific subgroup of patients—such as those with refractory epilepsy or severe neurological sequelae, for whom ambulances are frequently requested by their families due to recurrent seizures—may have contributed to the high standard deviation and mean. In addition, the high number of patients in the older adult age group in this study may be due to older adults living alone and experiencing difficulties in accessing hospital services. In addition, the high prevalence of chronic diseases among these patients further increases ambulance utilization. We believe that improving the provision of home care to older adults and facilitating outpatient appointment scheduling through home care services or family physicians would reduce ambulance utilization rates.

Ashburn et al. reported that a mean occupation time of ambulance teams at the scene of 14.2 min, a mean response time of 10 min, and a mean transport time of 17.5 min [11]. In the present study, the mean medical intervention time was 911.1 ± 712.5 s (15.2 min) and the mean occupation time was 50 min. These findings are consistent with the literature. It should be noted that patients who make frequent unnecessary requests for ambulances keep ambulance crews occupied for significant amounts of time, increasing the workload of the ambulance teams and prolonging the time it takes them to reach other patients.

In a study by Scott et al. of 100 patients who most frequently requested emergency ambulances, the most common calls were made for medical reasons [12]. In a study of patients who frequently used EMS conducted in Singapore, it was observed that only 10.7% were for trauma [13]. In the present study, similar to the literature, medical reasons were more common in both the adult and the pediatric age groups, while trauma patients accounted for only 7.6% in both groups.

In a study of patients who were not transported to hospital, it was found that 26.9% of patients who called for an emergency ambulance were not transported [14]. In the present study, this rate was 24.0%, similar to the literature. In a study of patients who made frequent use of EMS, most requests were made during the morning shift (59.1%) [9]. In our study, ambulances were dispatched patients most frequently between 08:00 and 15:59 (41.8%). In a study investigating frequent ambulance use, ambulance utilization was most common between 12:00 and 18:00, and 18:00 and 24:00 [15]. We believe that the different times of ambulance utilization across different countries may be related to differences in ease of access to healthcare services and associated barriers.

In the present study, no significant difference was observed in the frequency of ambulance use during the COVID-19 pandemic period (2020–2021) compared with other years. We believe that this may reflect the effectiveness of the quarantine measures implemented during the pandemic and the importance of preventing disease transmission.

### 4.1. Limitations

This study has several limitations. First, the ASOS database does not include data on social, economic, or chronic disease variables. Our analysis focused exclusively on ambulance utilization and did not capture patients’ use of primary care or outpatient hospital services. Consequently, the overall healthcare burden associated with these patients could not be comprehensively evaluated.

Additionally, due to the large sample size, statistical significance may not necessarily reflect clinical significance. The retrospective design of the study should also be noted as another potential limitation.

## 5. Conclusion

In the present study, frequent users were assigned an ambulance an average of 13.4 ± 26.1 times, and were most frequently ≥65 years of age. Each visit made by ambulance crews to frequent users lasted more than 50 min. The frequent use of ambulances by patients may be a factor in the delays experienced by ambulance teams in reaching other patients. To reduce ambulance utilization rates, chronic disease management programs should be strengthened, and home healthcare services should be made more effective. In addition, frequent users of ambulance services should be identified, case management programs should be implemented, and these patients should be followed up in outpatient settings. Finally, community-based preventive interventions should be developed to facilitate access to healthcare services for older adults, the disabled, and underserved populations.

## Figures and Tables

**Table 1:** Demographic characteristics of the patients.

Age (years)	59.0 ± 23.5*

Sex	
Female	50.2%
Male	49.8%

Age groups	
<65 years	49.8% (n = 140,445)
<18 years	5.5%
18–44 years	22.2%
45–64 years	22.1%
≥65 years	50.2 % (n = 141,698)

Nationality	
Turkish citizens	98.5% (n = 277,888)
Others	1.5% (n = 4255)

* Mean ± SD.

**Table 2:** Comparison of ambulance use and intervention times by age group.

	Value (mean ± SD)	p

Number of ambulance calls	13.4 ± 26.1	

<18 years	23.6 ± 61.5	<0.05
≥18 years	12.8 ± 22.2	

Intervention time (s)	911.1 ± 712.5	

<18 years	857.6 ± 654.2	<0.05
≥18 years	914.2 ± 796.3	

Occupation time (s)	3025.3 ± 1803.6	

<18 years	3476.1 ± 2285.3	<0.05
≥18 years	2998.9 ± 1767.9	

Distance (km)	18.1 ± 9.7	

<18 years	25.1 ± 24.2	<0.05
≥18 years	17.7 ± 19.3	

**Table 3:** Reasons for ambulance calls, diagnoses, and patient transfers.

			n (%)	n (%)		
Call reason		Age groups				
		Total n (%)	<18 years (n = 15,580) n (%)	≥18 years (n=266,563) n (%)		
	Medical	260,711 (92.4)	14,403 (92.4)	246,308 (92.4)		
	Other accidents	12,530 (4.4)	650 (4.2)	11,880 (4.5)		
	Stab/cut injuries	3669 (1.3)	247 (1.6)	3422 (1.3)		
	Traffic accidents	2433 (0.9)	114 (0.7)	2319 (0.9)		
	Suicide	2008 (0.7)	153 (1.0)	1855 (0.7)		
	Work accident	643 (0.2)	2 (0.0)	641 (0.2)		
	Fire	149 (0.1)	11 (0.1)	138 (0.1)		
Diagnosis					Adjusted OR (95% CI)	p*
	Dyspnea	26,560 (9.4)	717 (4.6)	25,843 (9.7)	2.86 (2.75–2.98)	<0.05
	COVID-19	16,726 (5.9)	415 (2.7)	16,311 (6.1)	1.80 (1.72–1.89)	<0.05
	Nausea and vomiting	14,235 (5.0)	859 (5.5)	13,376 (5.0)	2.21 (2.10–2.32)	<0.05
	Pain, unspecified	13,529 (4.8)	412 (2.6)	13,117 (4.9)	0.97 (0.93–1.01)	0.146
	Chest pain	12,157 (4.3)	205 (1.3)	11,952 (4.5)	8.27 (7.55–9.05)	<0.05
	Hypertension	10,923 (3.9)	41 (0.3)	10,882 (4.1)	1.25 (1.19–1.31)	<0.05
	Falls	8220 (2.9)	331 (2.1)	7889 (3.0)	2.43 (2.21–2.66)	<0.05
	Fever	7378 (2.6)	946 (6.1)	6432 (2.4)	1.33 (1.25–1.42)	<0.05
	Senility	6828 (2.4)	7 (0.0)	6821 (2.6)	0.34 (0.32–0.36)	<0.05
	Epilepsy	9152 (3.2)	1971 (12.7)	7181 (2.7)	1.03 (0.98–1.08)	0.228
	Others	165,501 (58.7)	9942 (63.8)	155,559 (58.4)		

* Multivariable logistic regression test.

**Table 4:** Hospitals to which patients were transferred.

		Total n (%)	<18 years (n = 15,580) n (%)	≥18 years (n = 266,563) n (%)
Result	Transferred to hospital	214,436 (76.0)	12,786 (82.1)	201,650 (75.6)
	* Tertiary hospital*	130,674 (60.9)	6576 (51.4)	124,098 (61.5)
	* Secondary hospital*	51,069 (23.8)	2093 (16.4)	48,976 (24.3)
	* University hospital*	24,606 (11.5)	3958 (31.0)	20,648 (10.2)
	* Private hospital*	8087 (3.8)	159 (1.2)	7928 (3.9)
	Refused transfer	64,628 (22.9)	2690 (17.3)	61,938 (23.2)
	Treated on scene	3079 (1.1)	104 (0.7)	2975 (1.1)

**Table 5:** Year, month, and time interval of most frequent ambulance calls by frequent users.

		Total n (%)	<18 years (n = 15,580) n (%)	≥18 years (n = 266,563) n (%)
Years	2018	56,269 (19.9)	3594 (23.1)	52,675 (19.8)
	2019	62,216 (22.1)	3993 (25.6)	58,223 (21.8)
	2020	54,457 (19.3)	2294 (14.7)	52,163 (19.6)
	2021	54,484 (19.3)	2874 (18.4)	51,610 (19.4)
	2022	54,717 (19.4)	2825 (18.1)	51,892 (19.5)
Months	January	24,406 (8.7)	1276 (8.2)	23,130 (8.7)
	February	22,457 (8.0)	1224 (7.9)	21,233 (8.0)
	March	24,547 (8.7)	1439 (9.2)	23,108 (8.7)
	April	23,507 (8.3)	1272 (8.2)	22,235 (8.3)
	May	23,748 (8.4)	1374 (8.8)	22,374 (8.4)
	June	23,072 (8.2)	1283 (8.2)	21,789 (8.2)
	July	23,991 (8.5)	1225 (7.9)	22,766 (8.5)
	August	23,666 (8.4)	1157 (7.4)	22,509 (8.4)
	September	22,484 (8.0)	1246 (8.0)	21,238 (8.0)
	October	23,874 (8.5)	1386 (8.9)	22,488 (8.4)
	November	22,664 (8.0)	1273 (8.2)	21,391 (8.0)
	December	23,727 (8.4)	1425 (9.1)	22,302 (8.4)
Time interval	00:00–07:59	52,623 (18.7)	2339 (15.0)	50,284 (18.9)
	08:00–15:59	118,005 (41.8)	7928 (50.9)	110,077 (41.3)
	16:00–23:59	111,515 (39.5)	5313 (34.1)	106,202 (39.8)
